# Temperature-Dependent Gentamicin Resistance of *Francisella tularensis* is Mediated by Uptake Modulation

**DOI:** 10.3389/fmicb.2016.00037

**Published:** 2016-01-28

**Authors:** Kathleen Loughman, Jesse Hall, Samantha Knowlton, Devin Sindeldecker, Tricia Gilson, Deanna M. Schmitt, James W.-M. Birch, Tara Gajtka, Brianna N. Kobe, Aleksandr Florjanczyk, Jenna Ingram, Chandra S. Bakshi, Joseph Horzempa

**Affiliations:** ^1^Department of Natural Sciences and Mathematics, West Liberty UniversityWest Liberty, WV, USA; ^2^Department of Microbiology and Immunology, New York Medical CollegeValhalla, NY, USA

**Keywords:** *Francisella*, gentamicin, antibiotic resistance, *Listeria monocytogenes*, *Klebsiella*, gentamicin uptake, temperature

## Abstract

Gentamicin (Gm) is an aminoglycoside commonly used to treat bacterial infections such as tularemia – the disease caused by *Francisella tularensis*. In addition to being pathogenic, *F. tularensis* is found in environmental niches such as soil where this bacterium likely encounters Gm producers (*Micromonospora* sp.). Here we show that *F. tularensis* exhibits increased resistance to Gm at ambient temperature (26°C) compared to mammalian body temperature (37°C). To evaluate whether *F. tularensis* was less permeable to Gm at 26°C, a fluorescent marker [Texas Red (Tr)] was conjugated with Gm, yielding Tr-Gm. Bacteria incubated at 26°C showed reduced fluorescence compared to those at 37°C when exposed to Tr-Gm suggesting that uptake of Gm was reduced at 26°C. Unconjugated Gm competitively inhibited uptake of Tr-Gm, demonstrating that this fluorescent compound was taken up similarly to unconjugated Gm. Lysates of *F. tularensis* bacteria incubated with Gm at 37°C inhibited the growth of *Escherichia coli* significantly more than lysates from bacteria incubated at 26°C, further indicating reduced uptake at this lower temperature. Other facultative pathogens (*Listeria monocytogenes* and *Klebsiella pneumoniae*) exhibited increased resistance to Gm at 26°C suggesting that the results generated using *F. tularensis* may be generalizable to diverse bacteria. Regulation of the uptake of antibiotics provides a mechanism by which facultative pathogens survive alongside antibiotic-producing microbes in nature.

## Introduction

Gentamicin (Gm) is an aminoglycoside antibiotic used to treat bacterial infections, including tularemia, a disease caused by *Francisella tularensis* (Alford et al., 1972; Jackson and Lester, 1978; Mason et al., 1980; Hassoun et al., 2006). This antibiotic binds to the bacterial 30S ribosomal subunit, which interferes with nascent polypeptide elongation (Mingeot-Leclercq et al., 1999). This binding impairs the machinery that prevents misreading of mRNA as well as premature termination of translation (Mingeot-Leclercq et al., 1999). In addition to being used clinically, Gm is also used in the “Gm protection assay” – a common laboratory test designed to quantify the survival and growth of intracellular pathogens in host cells (Isberg and Falkow, 1985; Hilbi et al., 2001; Horzempa et al., 2008b, 2011; Wisner et al., 2012; Schmitt et al., 2013).

Due to the highly polar nature of aminoglycosides, these antibiotics pass through the gram-negative outer membrane by disrupting Mg2+ bridges that stabilize the lipopolysaccharide molecules (Hancock, 1981; Mingeot-Leclercq et al., 1999). Import of aminoglycosides such as Gm through the cytoplasmic membrane is mediated through an energy-dependent process powered by proton motive force (PMF; Hancock, 1981; Taber et al., 1987). The specific cytoplasmic membrane transport machinery for Gm uptake has not yet been elucidated. Bacterial resistance to aminoglycosides can be facilitated by diminished permeability (Davies, 1971), and therefore understanding the molecular mechanism mediating Gm transport, and the regulation of this process is important.

As the body of an animal that died from tularemia decomposes, the *F. tularensis* bacteria are likely to inhabit the soil that absorbs the decaying material. Although *F. tularensis* has been identified in soil environments (Barns et al., 2005; Berrada and Telford, 2010; Broman et al., 2011), little is known about interactions with the microbiota, or how *F. tularensis* survives the chemical and physical stresses associated with a terrestrial inhabitance. For instance, the aminoglycoside Gm is naturally produced by the soil bacterium, *Micromonospora* sp. (Luedemann and Brodsky, 1963). Presumably, *F. tularensis* and other pathogenic bacteria that transiently occupy a soil niche would exhibit a means to survive this and other antibiotics produced by competing microorganisms prior to the colonization of a subsequent host.

Here we show that *F. tularensis* is more resistant to Gm at 26°C (a lower environmental temperature) than at 37°C (mammalian body temperature). This resistance is mediated by diminished antibiotic uptake at the lower temperature. Other pathogenic bacteria (*Listeria monocytogenes* and *Klebsiella pneumoniae*) exhibited similar enhanced resistance at 26°C, suggesting that the temperature-dependent uptake of Gm is generalizable to other microbes. We propose that bacteria that experience both a pathogenic and a temporary terrestrial life cycle evolved to utilize low temperature as a cue to decrease aminoglycoside uptake, increasing survival during habitation with competing bacteria that produce aminoglycosides and other antibiotics. During infection, these pathogenic bacteria inhabit an environment free of antibiotic-producing competing microbes (until antibiotics were introduced for clinical use approximately 75 years ago).

## Materials and Methods

### Bacterial Strains and Media

The strains of bacteria used in this study are listed in **Table 1**. *Francisella* bacteria cultivated on chocolate II agar (BD) were used to inoculate trypticase soy broth (TSB, BD) supplemented with 0.1% cysteine HCl (TSBc). *L. monocytogenes* bacteria cultivated on chocolate II agar were used to inoculate TSB supplemented with 0.6% yeast extract. *K. pneumoniae* bacteria were grown in TSB and *Escherichia coli* bacteria were cultured in LB broth. Broth cultures were incubated at 37°C with agitation. Kanamycin (Km) was included in the media at 10 μg/ml when necessary. All work with *F. tularensis* Schu S4 was conducted at the University of Pittsburgh in a biosafety level 3 facility with approval from the Centers for Disease Control and Prevention Select Agent Program.

**Table 1:** Bacterial strains used in this study.

Bacterial Strain	Reference
*Francisella tularensis* subsp. *holarctica* live vaccine strain	Karen Elkins
*F. tularensis* subsp. *tularensis* Schu S4 (NR-643)	NIH BEI Resources Repository^a^
*F. tularensis* LVS *emrA1*transposon mutant Km^R^	Ma et al., 2014
*F. tularensis* LVS *ΔpyrF*	Horzempa et al., 2010
*Escherichia coli* DH5α	Invitrogen
*F. novicida U112*	Karen Elkins
*Listeria monocytogenes* EGD	Douglas Drevets
*Klebsiella pneumoniae*	WLU-MCC^b^

*^a^National Institutes of Health Biodefense and Emerging Infections (NIH BEI) Research Resources Repository, National Institute of Allergy and Infectious Diseases.*

*^b^WLU-MCC, West Liberty University Microbiology Culture Collection, bacterial species routinely verified by standard metabolic and physiological tests.*

### Time-Kill Assays

Stationary phase broth cultures were suspended in phosphate buffered saline (PBS) or TSBc as indicated to a concentration of 2 × 10^8^ CFU/ml (*F. tularensis*) or 2 × 10^6^ CFU/ml (*L. monocytogenes* or *K. pneumoniae*). This phase was selected to minimize the effect of temperature on growth rate and metabolism of the bacteria (Horzempa et al., 2008a). Bacteria were normalized using OD_600_ and treated with 0, 50, 100, or 500 μg/ml Gm sulfate and incubated at 26 or 37°C. Antibiotic concentrations capable of showing significant bacterial killing over 2 h were selected. PABN (25 μM Phenylalanine-Arginine Beta-Naphthylamide) or CCCP (25 μM cyanide-m-chlorophenylhydrazone) was added when indicated. At the times indicated, suspensions of bacteria were serially diluted in PBS and plated to determine CFU.

### Antibiotic Disk Diffusion Assays

Stationary phase broth cultures were diluted to an optical density (*A*_600_) of 0.3. 100 μl of this suspension was spread plated onto solid medium (chocolate II agar for *F. tularensis*, LB agar for *E. coli*, trypticase soy agar for *K. pneumoniae*). Sterile Whatman filter disks infused with antibiotics were placed onto the surface of these plates that were subsequently incubated at 37°C overnight. The diameters of the zones of inhibition were measured using a metric ruler.

A variation of this disk diffusion assay was used to determine whether *F. tularensis* exhibited diminished uptake of Gm at 26°C. Here, *F. tularensis* LVS broth cultures were grown to stationary phase. Cultures were diluted to an optical density (*A*_600_) of 0.3 in TSBc and were incubated with 500 μg/ml Gm at 26 or 37°C. After 15 min, these bacteria were washed twice with PBS (each wash consisted of centrifuging the bacterial suspension at 16,300 × *g* for 3 min, and suspending the pellet in PBS). After the second wash, bacteria were centrifuged similarly and the pellet was suspended in 25 μl deionized water. This suspension was sonicated (3 s burst) to lyse cells. Lysates were added to a filter disk that was placed onto solid medium that had been spread plated with *E. coli.* These plates were incubated at 37°C overnight and subsequently, zones of inhibition were measured using a metric ruler.

### Uptake of Gm Conjugated to Texas Red

Texas Red^®^-X, succinimidyl ester (Life Technologies) was conjugated to Gm as previously described (Sandoval et al., 1998; Li et al., 2011). Briefly, a 300:1 molar ratio (Gm sulfate: Texas Red succinimidyl ester) were incubated together in K_2_CO_3_ (100 mM, pH = 10; Li et al., 2011). These conditions minimize the addition of multiple fluorescent moieties to a single Gm molecule and also ensure a minimal amount of free, unbound Texas Red (Sandoval et al., 1998; Li et al., 2011). Unbound Texas Red was removed using Pierce^TM^ Dye Removal Columns (Thermo Scientific) according to protocol of the manufacturer. Conjugation and removal of unbound Texas Red were verified by agarose gel electrophoresis (data not shown).

*Francisella tularensis* LVS bacteria were suspended in TSBc and treated with Tr-Gm (at an amount equivalent to 128 μg/ml Gm) at either 26 or 37°C. To measure bacterial fluorescence at the times indicated, *F. tularensis* cells were centrifuged at 16,300 × *g* for 3 min, the pellets were washed twice with PBS, and after an additional centrifugation were suspended in PBS. The fluorescence was determined using an Eppendorf plate reader using a 535/595 filter.

When indicated, bacteria were incubated with both Tr-Gm (at an amount equivalent to 128 μg/ml Gm) and increasing concentrations of Gm (0, 50, 100, 150, or 200 μg/ml) for 15 min at 37°C. Bacteria were centrifuged, washed twice in PBS, and then fluorescence was determined using an Eppendorf plate reader with a 535/595 filter.

### Statistical Analyses

Data were analyzed for significant differences using GraphPad Prism software. The statistical tests used are indicated in the figure legends.

## Results

**FIGURE 1:**
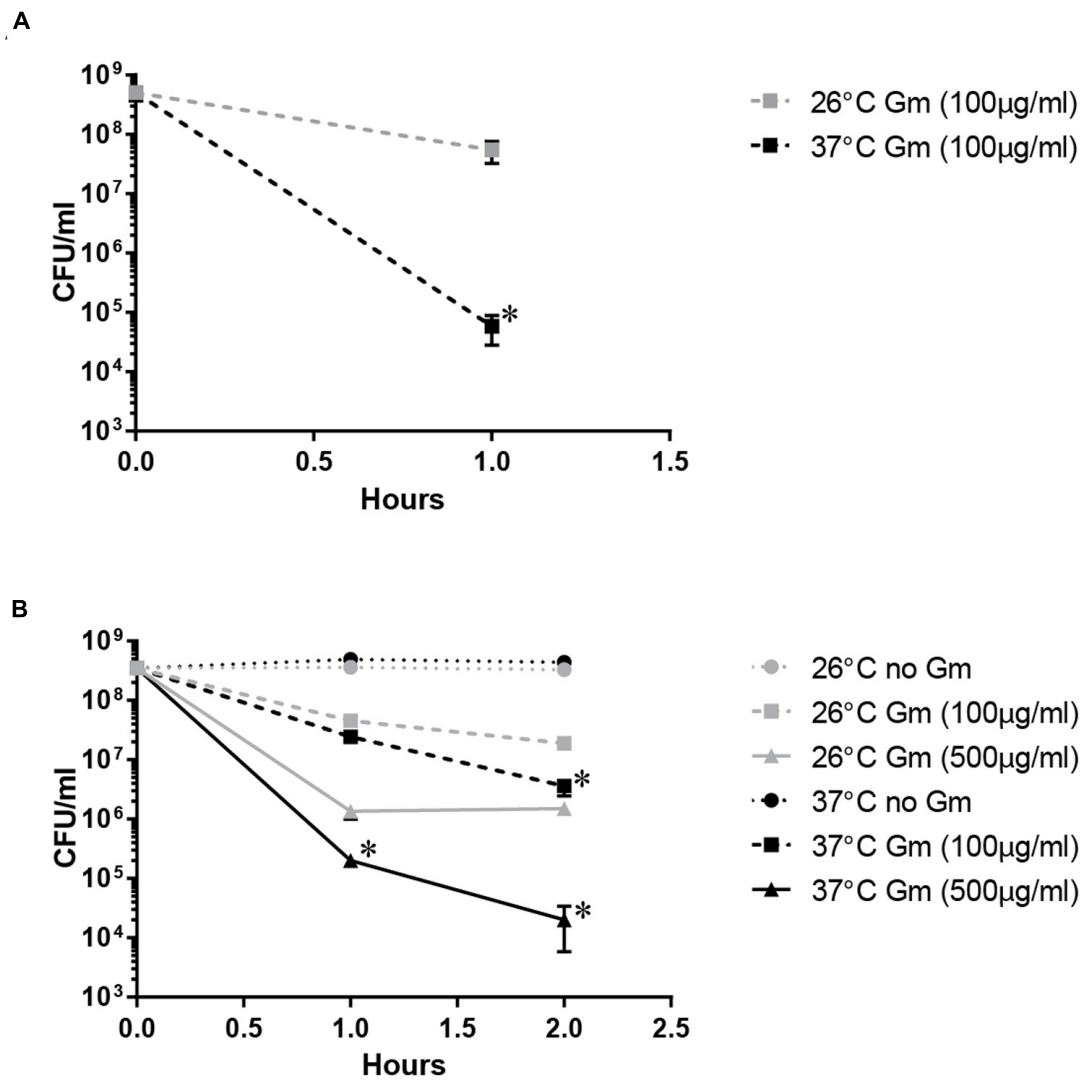
**Temperature-dependent Gm resistance of *Francisella tularensis*.** *F. tularensis* Schu S4 **(A)** and LVS **(B)** showed increased resistance to gentamicin (Gm) at ambient temperature (26°C) compared to mammalian body temperature (37°C). Bacteria were cultivated in general growth media, suspended in PBS, and treated with Gm at the indicated temperature. Bacteria were diluted and plated for viable CFU at the indicated times. Plotted values represent mean CFU ± SD. Log-transformed data were analyzed using a two-way ANOVA and a Sidak’s multiple comparisons test. ^∗^*P* < 0.05 Schu S4 1 h 26°C vs. 37°C **(A)**; LVS 1 h 26°C vs. 37°C Gm (500 μg/ml), 2 h 26°C vs. 37°C Gm (100 and 500 μg/ml; **B**).

The Gm protection assay is used to quantify uptake and intracellular replication of pathogenic bacteria (Isberg and Falkow, 1985; Horzempa et al., 2011). This method involves the utilization of Gm, an aminoglycoside that permeates poorly into eukaryotic cells, to kill extracellular bacteria. Cultured host cells that had been incubated with pathogenic bacteria for a period of time are treated with Gm, washed, lysed, and intracellular CFU are determined by plating serial dilutions. When working out the conditions for this assay using *F. tularensis*, we observed that the incubation temperature substantially affected the ability of Gm to reduce bacterial viability (**Figure 1**). Here, bacteria were incubated with Gm suspended in PBS and incubated at 37°C (mammalian body temperature) or at 26°C (ambient temperature). Incubation of *F. tularensis* Schu S4 with Gm (100 μg/ml in PBS) at 37°C for 1 h resulted in a 10,000-fold reduction of CFU, whereas incubation with this same antibiotic at 26°C only reduced CFU by 10-fold (**Figure 1A**). This indicated that the *F. tularensis* Schu S4 bacteria were more resistant to Gm at ambient versus mammalian body temperature. Incubation of *F. tularensis* Schu S4 with 20 μg/ml Gm for 1 h at either temperature did not result in a significant reduction of viability (data not shown). In addition, the attenuated *F. tularensis* LVS strain exhibited a similar phenotype with increased Gm resistance at 26°C (**Figure 1B**). Notably, fewer *F. tularensis* LVS bacteria were killed by the 100 μg/ml Gm than Schu S4, indicating an intrinsic disparity in Gm susceptibility between these strains, which is consistent with other published reports (Kreizinger et al., 2013). However, at both 100 and 500 μg/ml Gm, *F. tularensis* LVS bacteria appeared to be more resistant at the lower temperature (26°C) than at mammalian body temperature (**Figure 1B**).

**FIGURE 2:**
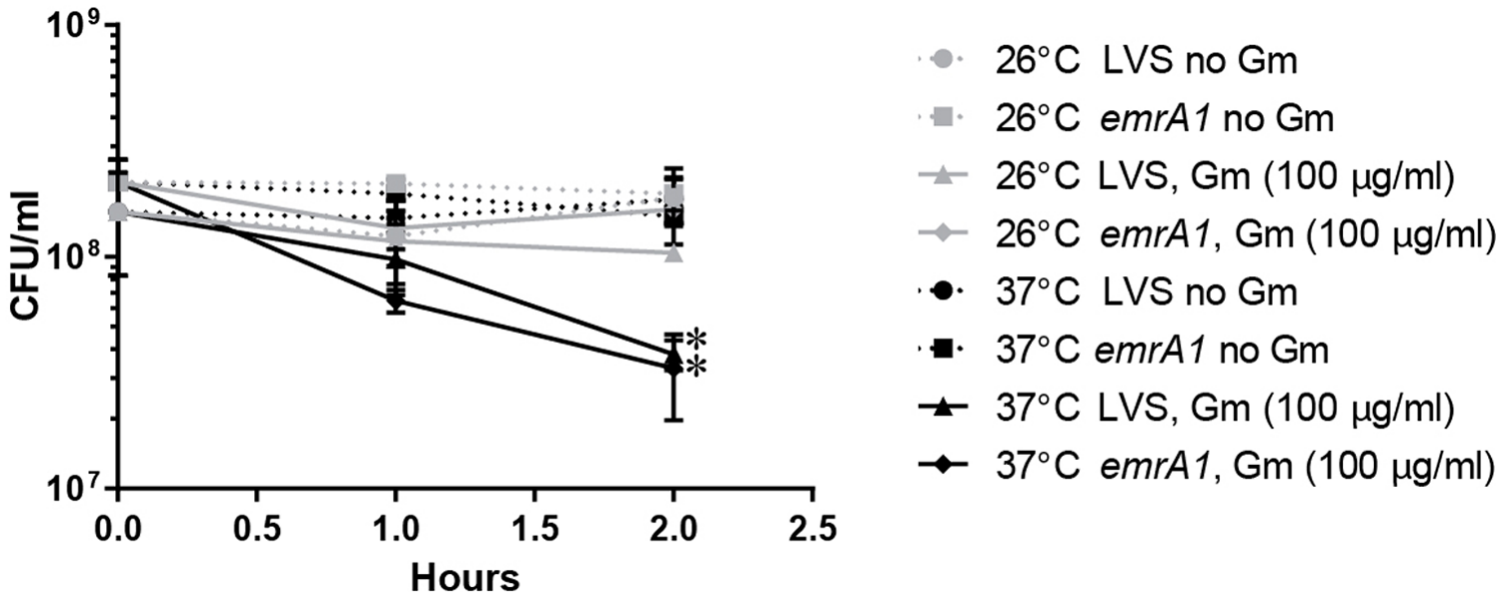
**Mutation of *emrA1* gene in LVS does not change this bacterium’s resistance to Gm at 26°C.** EmrA1 is a member of the membrane fusion protein family and is associated with the outer membrane TolC homolog SilC, to form a multidrug eﬄux pump. Increased Gm-resistance at 26°C is not mediated via eﬄux through the encoded eﬄux pump. Bacteria were suspended in PBS, and treated with Gm (100 μg/ml) for 2 h at the indicated temperature. Bacteria were diluted and plated for viable CFU at the times indicated. Plotted values represent mean CFU ± SD. Log-transformed data were analyzed using a two-way ANOVA and a Sidak’s multiple comparisons test. ^∗^*P* < 0.05 LVS 2 h 26 vs. 37°C Gm (100 μg/ml), *emrA1* 2 h 26°C vs. 37°C Gm (100 μg/ml). LVS vs. *emrA1* not significant.

Because the attenuated *F. tularensis* LVS also exhibited temperature-dependent Gm resistance, we utilized this strain to investigate the mechanism of this resistance throughout this study. Resistance to aminoglycosides can be manifested through drug inactivation, ribosomal alteration, antibiotic eﬄux, metabolic dormancy, or by diminished uptake (Mingeot-Leclercq et al., 1999; Allison et al., 2011). The genome of *F. tularensis* does not encode any proteins homologous to those known to inactivate aminoglycosides (data not shown). Moreover mutations that lead to modification of the ribosome structure are unlikely to cause this resistance at 26°C given the short duration of this assay and magnitude of surviving bacteria at 26°C compared to 37°C. Therefore, we first experimentally tested whether multi-drug eﬄux was enhanced at 26°C. EmrA1 comprises a component of a multidrug eﬄux pump of *F. tularensis* (Ma et al., 2014). This membrane fusion protein actively expels toxic materials, such as various types of antibiotics, from the bacterial cytosol (Ma et al., 2014). Similar to wild-type bacteria, the *F. tularensis emrA1* mutant was more resistant to Gm at 26°C compared to 37°C (**Figure 2**). This suggests that enhanced EmrA1-mediated eﬄux is not responsible for the increased resistance to Gm at 26°C.

**FIGURE 3:**
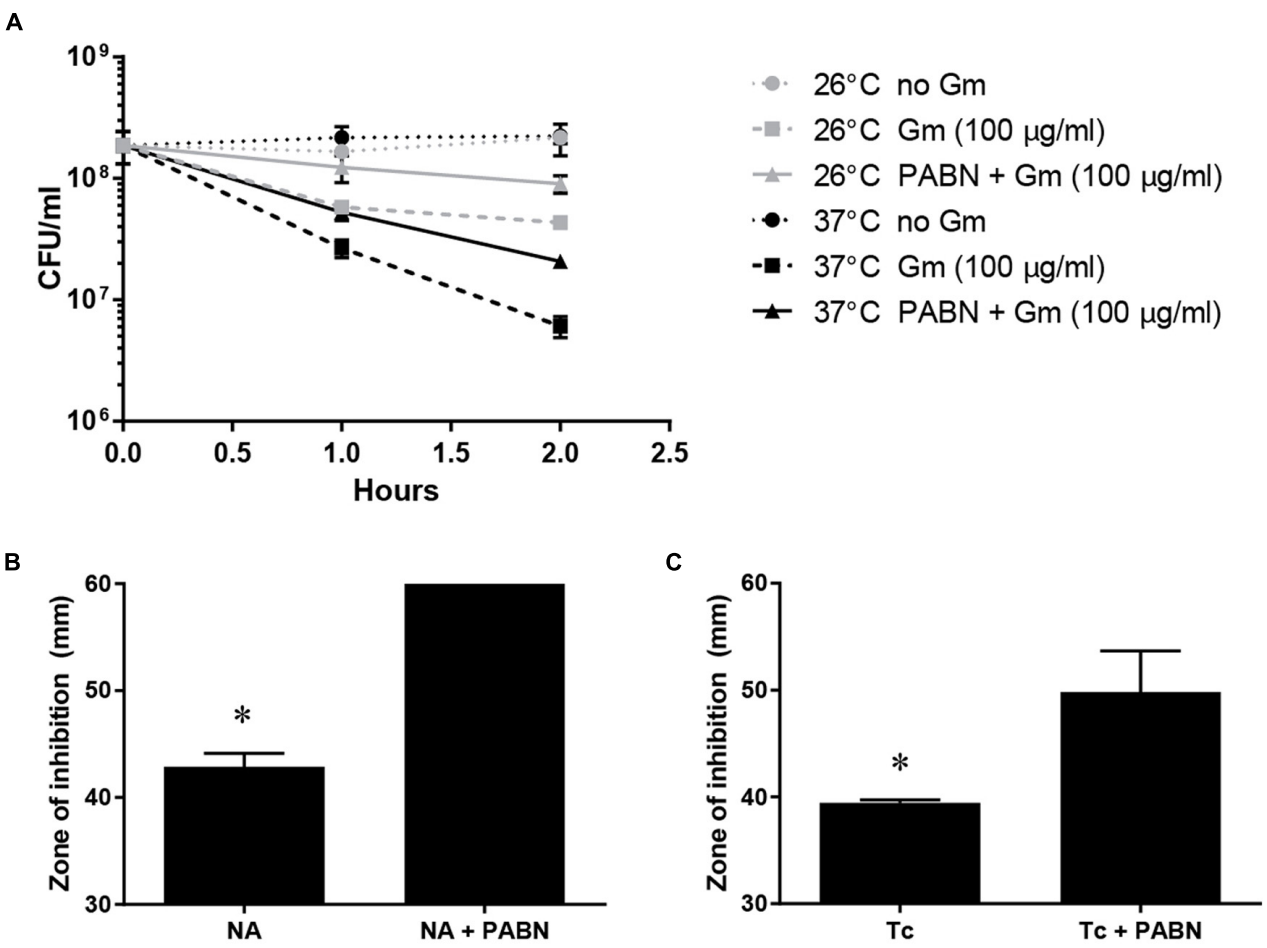
**Inhibition of multidrug eﬄux does not reduce the resistance of *F. tularensis* LVS to Gm at 26°C.** Bacteria were cultivated in general growth media, suspended in PBS, and treated with Gm, or Gm + PABN (25 μM Phenylalanine-Arginine Beta-Naphthylamide; eﬄux pump inhibitor) at the indicated temperature **(A)**. Bacteria were diluted and plated for viable CFU at the indicated times. Plotted values represent mean CFU ± SD. *F. tularensis* LVS bacteria were subjected to disk diffusion assays in the presence or absence of PABN to show that this compound was capable of inhibiting multi-drug eﬄux **(B,C)** as reported for other bacteria *F. tularensis* LVS is known to utilize multidrug eﬄux to expel nalidixic acid (NA) and tetracycline (Tc). Disks contained 30 μg of the indicated antibiotic. Bars represent the mean of the zones of inhibition ±SE. Media containing PABN significantly increased the zones of inhibition produced by NA (**B**; ^∗^*P* < 0.0001, unpaired *t*-test) and Tc (**C**; ^∗^*P* = 0.0271, unpaired *t*-test) likely due to reduced eﬄux activity.

To further investigate whether enhanced Gm eﬄux mediated the increased resistance at 26°C, bacteria were treated with PABN, a non-specific inhibitor against RND-type multidrug eﬄux pumps (Renau et al., 1999; Lamers et al., 2013; Opperman and Nguyen, 2015). Together with the PABN, these bacteria were treated with Gm, incubated at 26 or 37°C, and were then plated to determine viable CFU (**Figure 3A**). Inclusion of PABN did not reduce resistance at 26°C, providing further evidence that enhanced eﬄux at this lower temperature does not mediate the observed resistance. *F. tularensis* LVS has been shown to utilize multidrug eﬄux to expel nalidixic acid (NA) and tetracycline (Tc) (Forslund et al., 2006; Gil et al., 2006; Bina et al., 2008; Platz et al., 2010). Antibiotic disk diffusion assays indicated the inclusion of PABN increased the susceptibility of *F. tularensis* LVS to both NA (**Figure 3B**) and Tc (**Figure 3C**) – findings consistent with the inhibition of eﬄux machinery. These data verify that PABN was likely inhibiting antibiotic eﬄux machinery during the assay depicted in **Figure 3A**. Altogether, **Figures 2** and **3** indicate that the increased resistance to Gm at 26°C by *F. tularensis* is not likely mediated by enhanced eﬄux.

**FIGURE 4:**
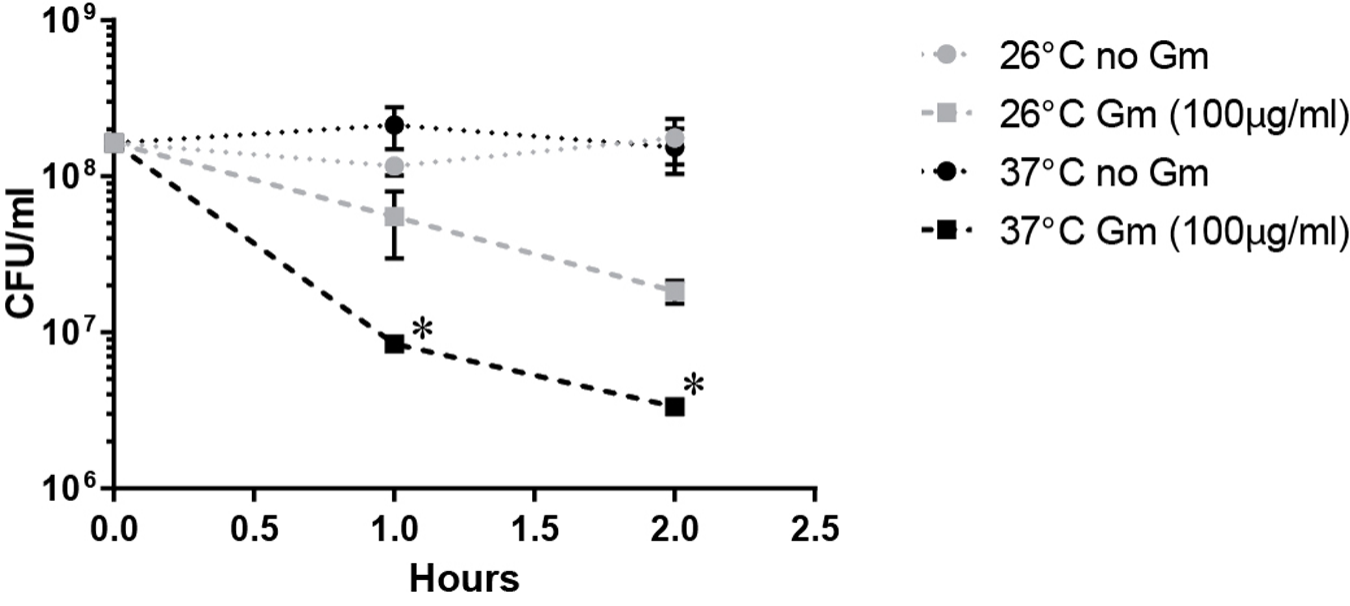
**Incubation in a rich medium maintains the observed Gm resistance at 26°C.** Bacteria were cultivated in general growth media, suspended in trypticase soy broth supplemented with 0.1% cysteine (TSBc), and treated with Gm at the indicated temperature. Bacteria were diluted and plated for viable CFU at the indicated times. Plotted values represent mean CFU ± SD. Log-transformed data were analyzed using a two-way ANOVA and a Sidak’s multiple comparisons test. ^∗^*P* < 0.05 LVS 26°C vs. 37°C Gm (100 μg/ml) at 1 and 2 h.

The observed increased resistance to Gm at 26°C was manifested in bacteria that were suspended in PBS resulting in starvation conditions. Because lack of nutrients can trigger tolerance to antibiotics (Nguyen et al., 2011), we hypothesized that the lower temperature enhanced the starvation response, increasing the number of bacteria surviving at 26°C. To test whether starvation conditions augmented resistance at 26°C, we incubated bacteria treated with Gm in TSBc – a rich medium routinely used for the cultivation of *F. tularensis*. Similarly to when bacteria were incubated in PBS, suspension in TSBc resulted in an increased resistance to Gm at 26°C compared to 37°C (**Figure 4**). This suggests that an enhanced starvation response at 26°C did not increase resistance to Gm.

**FIGURE 5:**
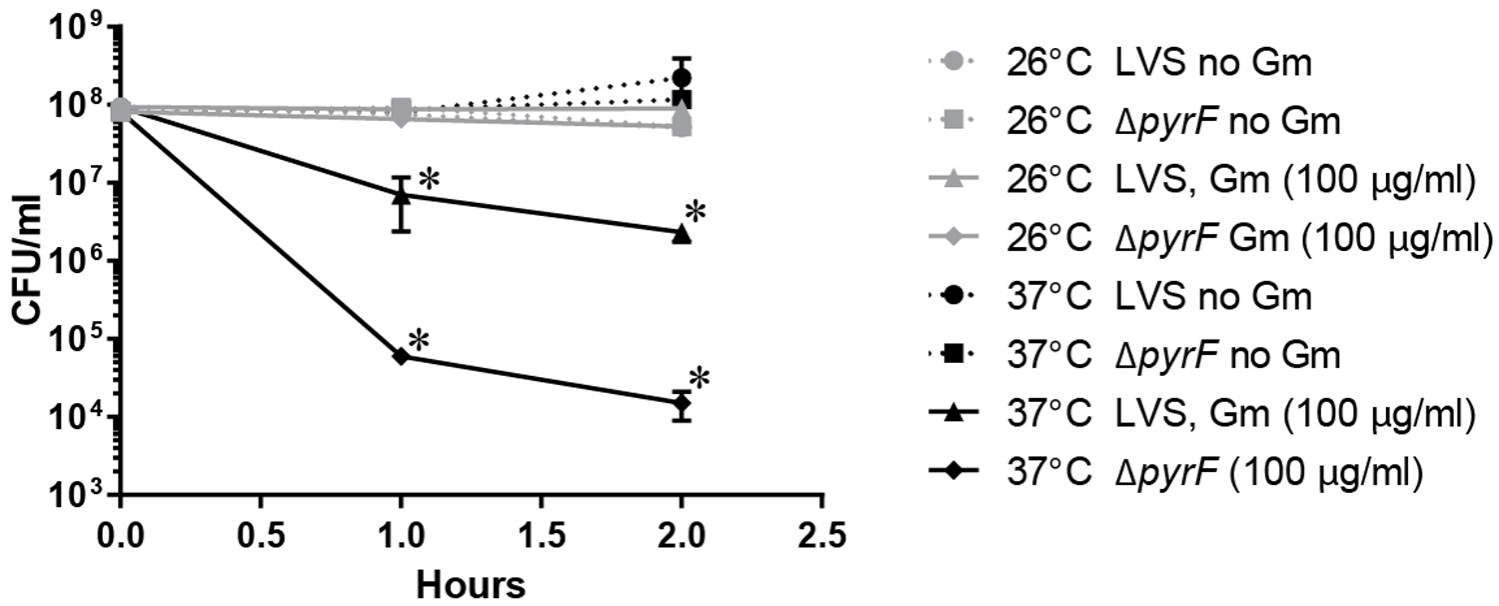
***Francisella tularensis*Δ*pyrF* is not more resistant to Gm than wild type LVS.** Bacteria were cultivated in general growth media, suspended in TSBc, and treated with Gm at the indicated temperature. Bacteria were diluted and plated for viable CFU at the indicated times. Plotted values represent mean CFU ± SD. Log-transformed data were analyzed using a two-way ANOVA and a Sidak’s multiple comparisons test. LVS 26°C vs. Δ*pyrF* Gm (100 μg/ml) not significant. ^∗^*P* < 0.05 LVS 26°C vs. 37°C Gm (100 μg/ml) at 1 and 2 h; ^∗^*P* < 0.05 LVS 37°C vs. Δ*pyrF* Gm (100 μg/ml) at 1 and 2 h.

To further assess whether a reduced metabolic state mediated the observed resistance at ambient temperature, we treated the *F. tularensis* LVS Δ*pyrF* mutant with Gm, incubated at 26 or 37°C, and determined the number of viable CFU (**Figure 5**). This strain is incapable of synthesizing uracil, and therefore grows at a reduced rate (Horzempa et al., 2010) which is indicative of a diminished metabolism. At 26°C, the *F. tularensis* LVS Δ*pyrF* bacteria were similarly resistant to Gm as wild type bacteria (**Figure 5**) indicating that a reduced metabolism was not responsible for the resistance to Gm at 26°C. Notably, the *F. tularensis* LVS Δ*pyrF* mutant bacteria were more sensitive to Gm than wild type bacteria at 37°C, indicating that a diminished metabolic capability seemingly increases sensitivity to this antibiotic under the conditions tested. Altogether, the results from **Figures 4** and **5** indicate that an enhanced starvation response at 26°C is not likely responsible for increasing *F. tularensis* resistance to Gm.

**FIGURE 6:**
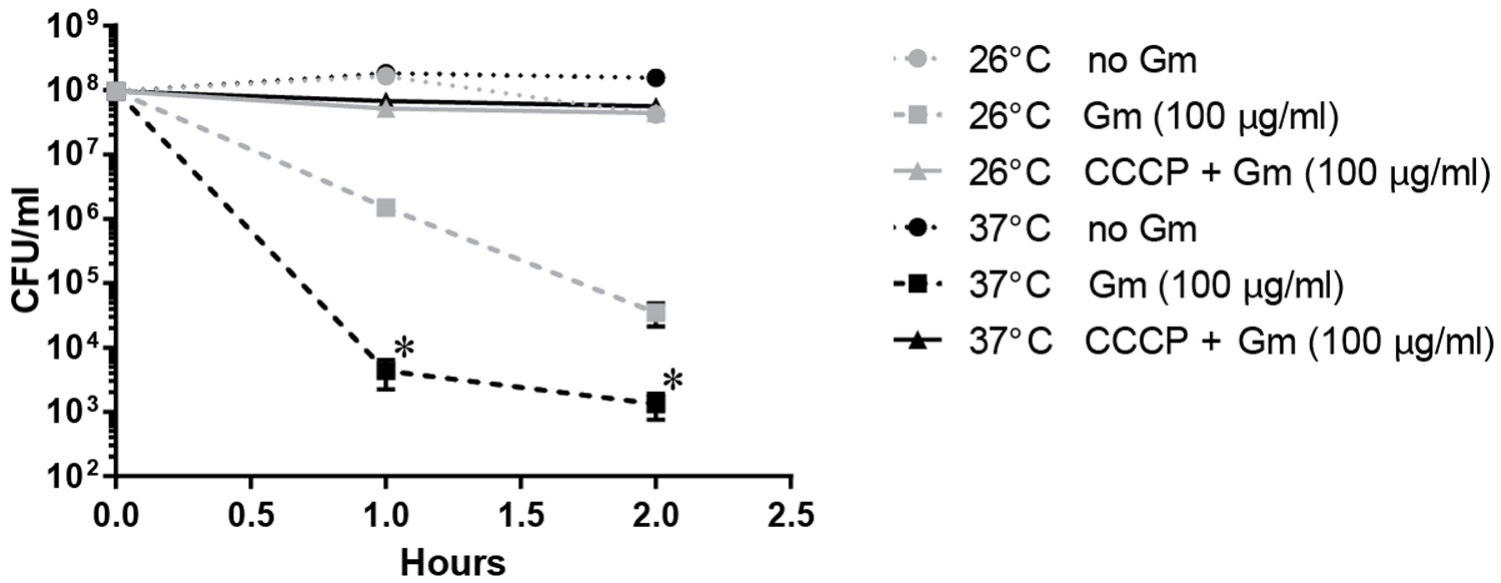
***Francisella tularensis* LVS takes up Gm using proton motive force (PMF) similarly to other Gram-negative bacteria.** Bacteria were cultivated in general growth media, suspended in TSBc, and treated with Gm, or Gm + CCCP (25 μM cyanide-m-chlorophenylhydrazone; a PMF inhibitor) at the indicated temperature. Bacteria were diluted and plated for viable CFU at the indicated times. Plotted values represent mean CFU ± SD. Log-transformed data were analyzed using a two-way ANOVA and a Sidak’s multiple comparisons test. ^∗^*P* < 0.05 LVS 26°C vs. 37°C Gm (100 μg/ml) at 1 and 2 h. LVS 26°C vs. 37°C CCCP + Gm (100 μg/ml) not significant.

We next sought to test whether decreasing uptake of Gm at 26°C mediated the observed resistance. Gram-negative bacteria import Gm utilizing PMF (Taber et al., 1987). Utilization of protonophores inhibit PMF, and thusly reduce bacterial uptake of aminoglycosides (Taber et al., 1987). To determine whether *F. tularensis* takes up Gm similarly to other Gram-negative bacteria, the protonophore, CCCP was used. Here, bacteria were treated with CCCP in combination with Gm, incubated at 26 or 37°C, and viable CFU were determined at the times indicated (**Figure 6**). The bacteria treated with CCCP survived Gm treatment, regardless of the incubation temperature, suggesting that this protonophore likely prevented uptake of Gm. This indicated that PMF mediates the uptake of Gm in *F. tularensis* similarly to other bacteria.

**FIGURE 7:**
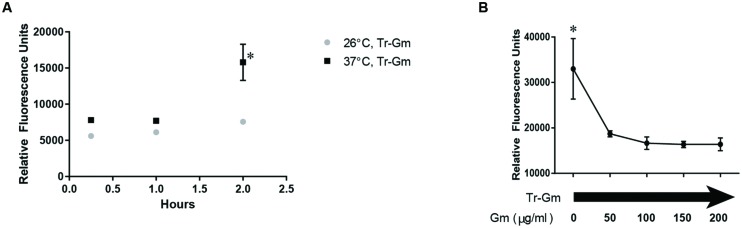
***Francisella tularensis* LVS takes up less Gm conjugated to Texas Red (Tr-Gm) at 26°C.** *F. tularensis* LVS showed an increase in Tr-Gm fluorescence at mammalian body temperature (37°C) compared to ambient temperature (26°) **(A)**. Bacteria were suspended in TSBc and treated with Tr-Gm (at an amount equivalent to 128 μg/ml Gm) at the indicated temperature. Bacteria were centrifuged, the pellets were washed twice and then suspended in PBS at the indicated times. The fluorescence was determined using an Eppendorf plate reader with a 535/595 filter. Plotted values represent mean relative fluorescence units ±SE. Data were analyzed using a two-way ANOVA and a Sidak’s multiple comparisons test. ^∗^*P* < 0.05 37°C vs. 26°C at 2 h; 37°C at 2 h vs. 37°C 15 min or 1 h. 26°C 2 h vs. 26°C 15 min or 26°C 1 h, not significant. To show that Tr-Gm was taken up through the Gm transporter **(B)**, bacteria were incubated with Tr-Gm and increasing concentrations of Gm for 15 min at 37°C. Bacteria were centrifuged, washed in PBS, and then fluorescence was determined using an Eppendorf plate reader with a 535/595 filter. Plotted values represent mean relative fluorescence units ±SE. Data were analyzed using a one-way ANOVA and a Dunnetts’s multiple comparisons test. ^∗^*P* = 0.0150, 0 μg/ml vs. all other conditions.

To determine whether *F. tularensis* took up less Gm at 26°C, we fluorescently labeled Gm with Texas Red (Tr-Gm) and subsequently analyzed cellular fluorescence similarly to previous investigations (Sandoval et al., 1998; Steyger et al., 2003; Allison et al., 2011). *F. tularensis* LVS bacteria were treated with Tr-Gm, incubated at 26 or 37°C, and at the indicated times, were washed three times with PBS, and analyzed for red fluorescence using a plate reader (**Figure 7A**). At each time point, bacteria incubated at 37°C were more fluorescent than those incubated at 26°C (**Figure 7A**). This suggested that at the lower temperature, bacteria took up less Tr-Gm. Inclusion of unconjugated Gm competitively inhibited uptake of Tr-Gm (**Figure 7B**). This indicated that the fluorescently labeled form of this antibiotic was taken up similarly to the unmodified Gm. These data indicate that the increased resistance of *F. tularensis* to Gm at 26°C (**Figures 1** and **4**) was likely due to diminished uptake of this antibiotic.

**FIGURE 8:**
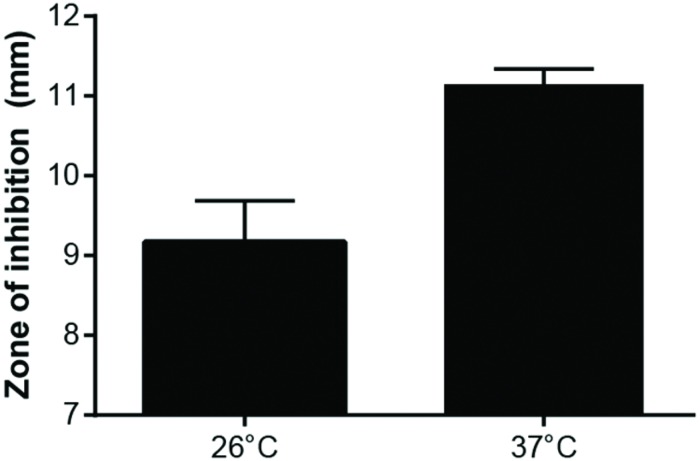
**Growth of *Escherichia coli* is inhibited more by *F. tularensis* LVS lysates from bacteria incubated with Gm at 37°C.** *F. tularensis* were suspended in TSBc and incubated with 500 μg/ml Gm at 26°C or 37°C. Subsequently, these bacteria were washed twice with PBS, then suspended in deionized water and sonicated for 3 s to lyse. Lysates were added to a filter disk that was placed onto a lawn-streaked agar plate of *E. coli.* These plates were incubated at 37°C overnight. *E. coli* grew up to the filter disk on plates containing *F. tularensis* LVS lysates from bacteria that were incubated in TSBc at 26°C and 37°C without Gm (not shown). Bars represent the mean of the zones of inhibition ±SE. *p* = 0.0114, unpaired *t*-test.

To confirm that *F. tularensis* took up less Gm at 26°C compared to 37°C, *F. tularensis* LVS bacteria were incubated with Gm at 26 or 37°C, and after a short period of time (15 min), these cells were washed twice with PBS, suspended in deionized water, and then lysed. Lysates were added to filter disks that were placed onto LB agar plates that had been lawn-streaked with *E. coli*. After incubation, zones of inhibition were measured. Disks infused with lysates of LVS bacteria that had been treated with Gm and incubated at 37°C were significantly larger than those produced by lysates of antibiotic-treated bacteria incubated at 26°C (**Figure 8**). Notably, lysates from bacteria not treated with antibiotic did not produce zones of inhibition (data not shown). This experiment confirms that the increased resistance to Gm at 26°C is due to diminished uptake by *F. tularensis* LVS.

**FIGURE 9:**
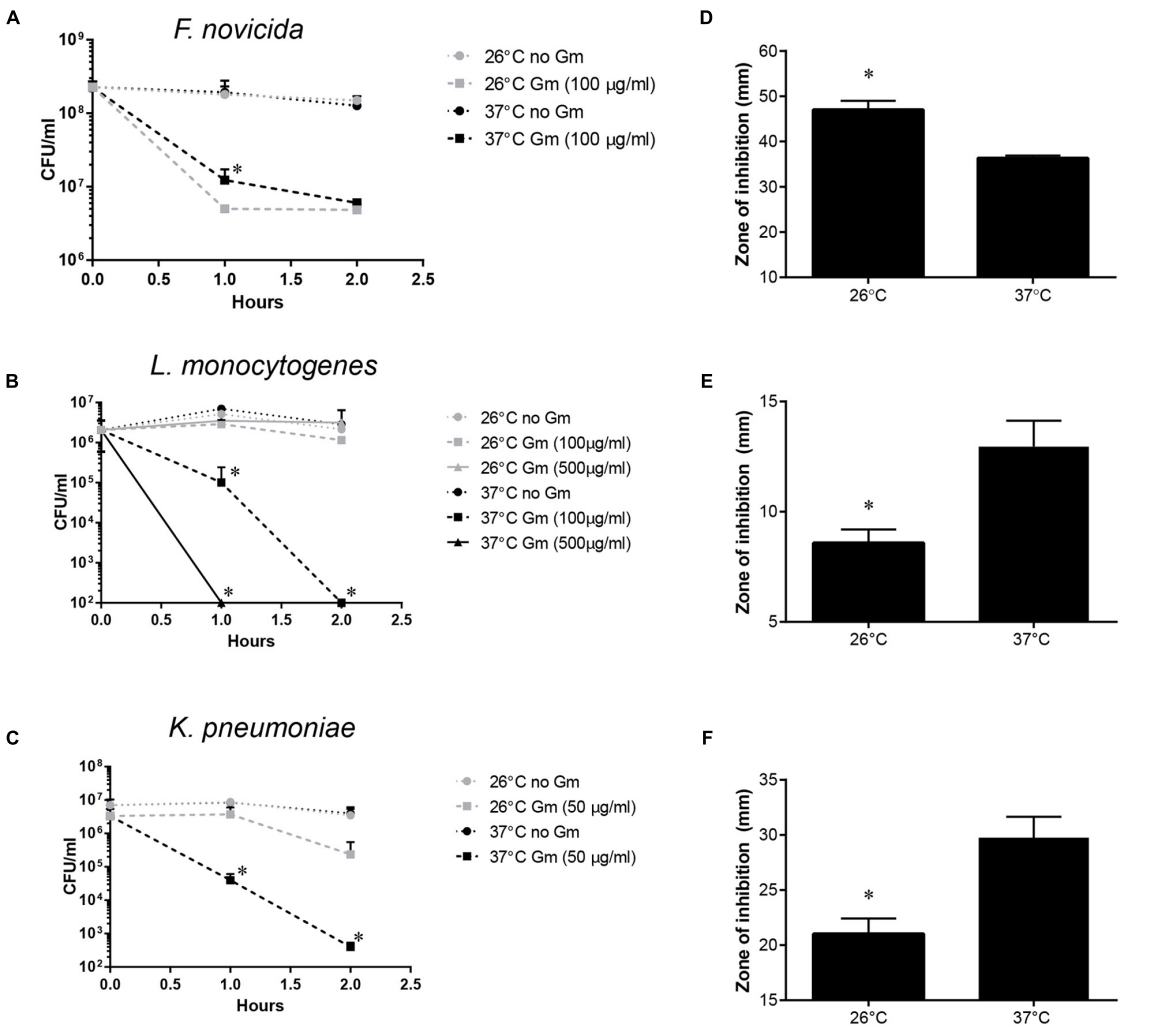
***Francisella* novicida, *Listeria monocytogenes* and *Klebsiella pneumoniae* exhibit temperature-dependent Gm resistance.** *F. novicida* **(A)**, *L. monocytogenes* **(B)**, or *K. pneumoniae* **(C)** bacteria were cultivated in general growth media overnight, suspended in PBS, and treated with Gm at the indicated temperature. Bacteria were diluted and plated for viable CFU at the indicated times. Plotted values represent mean CFU ± SD. Log-transformed data were analyzed using a two-way ANOVA and a Sidak’s multiple comparisons test. ^∗^*P* < 0.05 *F. novicida* 26°C vs. 37°C Gm (100 μg/ml) at 1 h **(A)**; *L. monocytogenes* 1 h 26°C vs. 37°C Gm (100 and 500 μg/ml), 2 h 26°C vs. 37°C Gm (100 and 500 μg/ml); *K. pneumoniae* 26°C vs. 37°C Gm (50 μg/ml) at 1 and 2 h. Antibiotic disk diffusion assays were carried out in which *F. novicida* **(D)**, *L. monocytogenes* **(E)**, or *K. pneumoniae* **(F)** bacteria were lawn streaked onto solid media, and incubated with filter disks containing the indicated amount of Gm. These plates were incubated at the temperature indicated, and zones of inhibition were measured. Bars represent the mean of the zones of inhibition ±SD. Disk diffusion data were analyzed using an unpaired *t*-test. ^∗^*P* = 0.0009 **(D)**; ^∗^*P* = 0.0026 **(E)**; ^∗^*P* = 0.0050 **(F)**.

Determining whether other bacteria possess increased Gm resistance at lower ambient temperature could provide further insight into the mechanism and the prevalence of this phenomenon. We therefore tested the impact of temperature on the Gm resistance of *F. novicida*, a bacterium related to *F. tularensis* that has been isolated from saltwater and rarely from human infections (Whitehouse et al., 2012; Kingry and Petersen, 2014). *F. novicida* bacteria were exposed to Gm followed by incubation at 26 or 37°C. Unlike *F. tularensis, F. novicida* showed a slight, but significantly increased resistance to Gm at 37°C 1 h after exposure (**Figure 9A**). However, after 2 h, bacteria incubated at either temperature were equally susceptible (**Figure 9A**). These data suggest that although genetically similar, *F. tularensis* and *F. novicida* regulate the uptake of Gm differently. We subsequently tested whether diverse bacteria exhibited increased Gm resistance at lower ambient temperatures similar to *F. tularensis. L. monocytogenes* is an intracellular pathogen that has been isolated from environmental sources. However, this bacterium is gram-positive (Firmicutes) indicating a great deal of taxonomic separation from *F. tularensis.* Similarly to *F. tularensis*, *K. pneumoniae* is a gram-negative γ-proteobacterium. However, *K. pneumoniae* is an opportunistic pathogen that is not associated with an intracellular lifestyle. Both *L. monocytogenes* (**Figure 9B**) and *K. pneumoniae* (**Figure 9C**) exhibited increased resistance to Gm at 26°C suggesting that diverse bacteria have developed a temperature-dependent resistance to this antibiotic. To confirm these findings, we conducted antibiotic disk diffusion assays in which plates that had been lawn-streaked with bacteria were incubated at 37 or 26°C. Incubation at the lower temperature yielded inconsistent and sparse growth of *F. tularensis* LVS, therefore negating the possibility of using this confirmatory test (data not shown). However, antibiotic disk diffusion assays for *F. novicida*, *L. monocytogenes*, and *K. pneumoniae* mirrored the time kill assays at 1 h post-exposure to Gm (**Figures 9D–F**). In other words, *F. novicida* was more resistant to Gm at 37°C, while *L. monocytogenes* and *K. pneumonaie* bacteria were more resistant at 26°C.

## Discussion

Data presented here indicated that *F. tularensis* and other bacteria that exist as facultative pathogens exhibit increased resistance to Gm at a lower temperature associated with environmental niches, versus mammalian body temperature. Evidence indicated that this increased resistance was due to diminished permeability to Gm at the lower temperature. This work describes a mechanism in which an important pathogen interacts with its non-host environment. *F. tularensis* has been identified in various environmental niches, including the soil (Barns et al., 2005; Berrada and Telford, 2010; Broman et al., 2011) – an environment that also harbors antibiotic producers. One such microbe, *Micromonospora*, produces Gm (Luedemann and Brodsky, 1963; Jao and Jackson, 1964). We propose here that *F. tularensis* and other pathogenic microbes have evolved general mechanisms to resist antibiotics during their existence outside of hosts. And, as demonstrated by using *K. pneumoniae* and *L. monocytogenes*, these bacteria use temperature as a cue to decrease permeability to antibiotics such as aminoglycosides. Previously published data indicated that transcription of a *F. tularensis* β-lactamase gene is induced at 26°C vs. 37°C (Horzempa et al., 2008a). We have since confirmed that *F. tularensis* β-lactamase activity is more robust at 26°C (data not shown) indicating that lower environmental temperature induces general resistance against many types of antibiotics. Largely throughout the course of evolution (prior to the clinical use of antibiotics in the 1940s), these mechanisms were not necessary during mammalian infection as niches occupied here are devoid of antibiotic-producing microbes.

The concentration of Gm used in many of the studies represented in this manuscript are relatively high compared to clinical use and soil concentrations which reach ∼mg/kg quantities (Kümmerer, 2008). However, *Micromonospora* sp. bacteria are capable of producing concentrations of Gm over 7 g/l in culture (Meenavilli et al., 2008) indicating that in soil microenvironments adjacent to these antibiotic-producing microbes, quantities could reach or even exceed levels similar to those used in the studies outlined here.

This study models interactions that *F. tularensis* would have with its environment during and following the decomposition of an animal that perished as a result of tularemia. In this soil environment (Barns et al., 2005; Berrada and Telford, 2010; Broman et al., 2011), *F. tularensis* is at some point likely to encounter antibiotic-producing microbes. As this study presented evidence that *F. tularensis* bacteria take up less aminoglycosides at lower temperatures, future investigations should focus on whether lower temperatures cue this bacterium to restrict uptake of other antibiotics associated with soil inhabitation, such as Tc. Little is known of the duration *F. tularensis* exists outside of a host cell or organism, or whether *F. tularensis* grows free from a host. Certainly, *F. tularensis* is capable of being cultivated in the laboratory in culture media devoid of host cells. However, due to the fastidious nature of this organism, it is unlikely capable of replication in the absence of essential host factors. Therefore, the increased resistance to Gm at lower environmental temperatures demonstrated here may provide an advantage until *F. tularensis* is phagocytosed or engulfed by a protozoan or arthropod host (Abd et al., 2003; Mahajan et al., 2011; Ozanic et al., 2015) – a niche inhabited with a lower abundance of antibiotic-producing microbes.

Diminished uptake of Gm at 26°C correlated with the increased resistance of *F. tularensis* LVS at this lower temperature, associating these two phenomena. Because a genetically similar bacterium, *F. novicida* did not exhibit the same pattern of resistance, increased membrane fluidity at the higher temperature was not likely responsible for the disparity in Gm uptake. An alternative explanation would be differential activity of a Gm transporter. Identification of the protein responsible for aminoglycoside uptake in bacteria has been elusive. Perhaps this is because many different proteins are capable of non-specifically importing this category of antibiotics. A current focus of our laboratory is to identify *F. tularensis* proteins responsible for the import of Gm.

Lipopolysaccharide (LPS) structure substantially affects the permeability of bacteria to polar molecules (Nikaido, 2003). Temperature greatly influences the structure of the lipid A moiety of the *Francisella* LPS molecule (Shaffer et al., 2007; Li et al., 2012). Therefore, it is possible that the addition of the 3-OH C16 acyl group at the lower environmental temperature could be responsible for the decreased permeability to Gm. Investigations to test this possibility are ongoing. However, *L. monocytogenes* (a gram-positive bacterium) also exhibited increased resistance to Gm at 26°C. This indicates that LPS modification in response to temperature cues could not be the mechanism utilized under all circumstances. Further testing will focus on determining the extent of resistance mechanisms utilized to resist aminoglycosides in response to lower environmental temperatures.

Although data presented here indicated that the temperature-dependent Gm resistance of *F. tularensis* is mediated by uptake modulation, we cannot completely rule out the possibility that this resistance is, at least in part, due to enhanced eﬄux of aminoglycosides. In *Pseudomonas aeruginosa*, the MexXY multidrug eﬄux system is one of the primary determinants of aminoglycoside resistance (Morita et al., 2012). Although PABN is a non-specific inhibitor of RND-type eﬄux pumps, this compound antagonized the activity of aminoglycosides in a MexXY-dependent manner in *P. aeruginosa* (Mao et al., 2001). If *F. tularensis* possesses a homolog of the MexXY multidrug eﬄux system or a functional equivalent, this machinery may contribute to the temperature-dependent resistance observed here.

An unanswered question raised by the data presented here is: why have *F. tularensis* LVS and other pathogenic bacteria evolved to import more Gm at mammalian body temperature? We speculate that this is a consequence of an overall upregulation of uptake machinery that occurs in response to host temperature (Horzempa et al., 2008a). We hypothesize that the Gm enters the cell non-specifically through nutrient uptake transporters during this general upregulation. This hypothesis is currently being tested in our laboratory.

This work presented a novel observation – that *F. tularensis* and other facultative pathogenic bacteria are more resistant to Gm at lower ambient temperatures (26°C) compared to mammalian body temperature (37°C). We show that this resistance was mediated by diminished antibiotic uptake at the lower temperature. We hypothesize that bacteria experiencing both a pathogenic and a temporary terrestrial life cycle may have evolved to utilize low temperature as a cue to decrease aminoglycoside uptake, increasing survival during habitation with competing bacteria that produce aminoglycosides.

## Author Contributions

JoH conceived and designed the experiments. KL, JeH, SK, DS, TrG, DMS, JB, TaG, BK, AF, JI, CB, and JoH constructed the mutants and performed the experiments. JoH, KL, DMS, and CB analyzed the data. JoH wrote the paper. JoH, KL, DMS, CB, and TaG edited the manuscript.

## Conflict of Interest Statement

The authors declare that the research was conducted in the absence of any commercial or financial relationships that could be construed as a potential conflict of interest.
